# Rigor, Reproducibility, and Responsibility (the 3 R’s) in the Scientific Publishing Landscape: ASM’S Pre-Publication Proactive Approaches

**DOI:** 10.1128/msphere.00598-22

**Published:** 2023-01-10

**Authors:** Amy L. Kullas

**Affiliations:** a Journals Department, American Society for Microbiology, Washington, DC, USA

**Keywords:** integrity, publishing ethics, rigor

## EDITORIAL

It is imperative that all stakeholders (e.g., researchers, consultants, contractors, funding agencies, institutions, professional societies, scholarly publishers, etc.) in the scientific ecosystem recognize that is time for a collaborative stance and shared responsibility for upholding research and data integrity. Although the research funding and landscape continues to intensify, there has been more of a focus on the overall rigor of science.

Recognizing its responsibility as a scientific society and scholarly publisher, the American Society for Microbiology (ASM) and American Academy of Microbiology (AAM) organized a colloquium and published a report entitled “Promoting Responsible Scientific Research” (https://asm.org/Reports/Promoting-Responsible-Scientific-Research). The report noted three main reasons for published research not being reproducible: selection and experimental bias, sloppy science, and scientific misconduct. Accordingly, ASM has implemented proactive approaches for reviewing and screening manuscripts prior to their publication. Before publishing a paper, ASM analyzes the figures for potential data manipulation as well as plagiarism/self-plagiarism concerns. ASM found that this increased prescreening of manuscripts has helped to reduce the number of problematic figures published within ASM’s journals. Thus, ASM is motivated to be a leader in publishing ethics within the scholarly publishing community.

Furthermore, the ASM Journals program has developed a robust Publishing Ethics Checklist (https://journals.asm.org/ethics-checklist), offering guidance on how authors can avoid common publishing ethics situations: avoiding duplicate submissions and publications, plagiarism/self-plagiarism, and inappropriate image manipulation. In late 2019, ASM expanded its Open Data policy (https://journals.asm.org/open-data-policy) to all of its journal titles, leading to an increase in the rigor and transparency of published data. Recent efforts to improve overall rigor and reproducibility include the ASM Journals anti-scooping policy (https://journals.asm.org/scooping-policy) and relaunching of *Microbiology Spectrum* to publish negative data studies, confirmatory or replication studies, and studies which have technically robust data that may contradict earlier studies (https://journals.asm.org/journal/spectrum/scope). As a scientific publisher, ASM continues to grow and strengthen its resources and programs to promote a culture of integrity and scientific rigor in the microbial sciences.

In a New York Times Opinion Editorial published in October 2022 (https://www.nytimes.com/interactive/2022/10/29/opinion/science-fraud-image-manipulation-photoshop.html?smtyp=cur&smid=tw-nytopinion), Dr. Elisabeth Bik describes how “science has a nasty photo-shopping problem” and provides examples of images where there may be “an intention to mislead.” Although the editorial notes that “peer review is not set up to detect fraud,” ASM works to educate the microbial sciences and the larger STEM community on issues of misconduct so that we can work toward a culture of rigor, reproducibility, and transparency. For example, ASM has piloted a couple of programs, such as *mBio* Junior Editors and *Microbiology Spectrum*’s Review Editors, to train scientists to be credible and thoughtful peer reviewers to deliver appropriate feedback to authors. Fittingly, publishing ethics is an important and informative module that often allows for engaging conversations.

ASM’s priority is to preserve the integrity of the scientific record. When ASM is contacted by a ‘concerned reader,’ including those that wish to remain anonymous, with potential figure concerns in a published paper in one of its journals, ASM staff independently review the allegations and, if the concern is substantiated, ASM follows its Publishing Ethics Policies and Procedures (https://journals.asm.org/publishing-ethics-procedures) for responding to and handling these allegations. Briefly, the respective journal’s ethics panel reviews the information of the case, and the editor-in-chief sends a formal inquiry to the corresponding author of the paper outlining the concerns and requesting an explanation along with the original, underlying data. In cases of potential fraud or research misconduct, the ethics panel will notify the authors’ institution(s) and request its assistance in reviewing and investigating the allegations, although these investigations can take time. ASM abides by the recommendations which result from the investigation.

It is also critical for the larger scientific community to take steps to prevent sloppy science. First, experiments should also be repeated to verify their reproducibility. The results should include appropriate statistics. Results from an experiment that has only been performed once are anecdotal and not scientific evidence. Additionally, it is important to retain original, unmodified images (such as blots, gels, microscopy, etc.) and ensure that these original images are labeled appropriately. Some authors also take a proactive approach toward transparency by depositing these original images in a repository or even publishing them as supplemental material. Helpful tips include saving versions of data at each modification, from raw data to figures prepared for publications, and noting specific data that have been included in your manuscripts. Lastly, take care to avoid “copy and pasting” errors when organizing figure panels.

Appreciating that there are opportunities and areas to grow, ASM continuously strives to implement new initiatives to enhance scientific and publishing integrity. The scientific publishing landscape is constantly evolving. Therefore, as scientists, it is important for us to ensure that accurate, reliable information is disseminated into society so that there continues to be trust and transparency in science, because when this does not happen, everyone can suffer.

